# Enhancing 3-hydroxypropionic acid production in combination with sugar supply engineering by cell surface-display and metabolic engineering of *Schizosaccharomyces pombe*

**DOI:** 10.1186/s12934-018-1025-5

**Published:** 2018-11-13

**Authors:** Seiya Takayama, Aiko Ozaki, Rie Konishi, Chisako Otomo, Mayumi Kishida, Yuuki Hirata, Takuya Matsumoto, Tsutomu Tanaka, Akihiko Kondo

**Affiliations:** 1 Department of Chemical Science and Engineering, Graduate School of Engineering, Kobe University, 1-1 Rokkodaicho, Nada, Kobe 657-8501 Japan; 2 Graduate School of Science, Technology and Innovation, Kobe University, 1-1 Rokkodaicho, Nada, Kobe 657-8501 Japan

**Keywords:** *Schizosaccharomyces pombe*, 3-Hydroxypropionic acid, CRISPR–Cas9 system, Cell surface display, Malonyl-CoA

## Abstract

**Background:**

Economical production of value-added chemicals from renewable biomass is a promising path to sustainability. 3-Hydroxypropionic acid (3-HP) is an important chemical for building a bio-sustainable society. Establishment of 3-HP production from renewable resources such as glucose would provide a bio-sustainable alternative to the production of acrylic acid from fossil resources.

**Results:**

Here, we describe metabolic engineering of the fission yeast S*chizosaccharomyces pombe* to enhance 3-HP production from glucose and cellobiose via the malonyl-CoA pathway. The *mcr* gene, encoding the malonyl-CoA reductase of *Chloroflexus aurantiacus*, was dissected into two functionally distinct fragments, and the activities of the encoded protein were balanced. To increase the cellular supply of malonyl-CoA and acetyl-CoA, we introduced genes encoding endogenous aldehyde dehydrogenase, acetyl-CoA synthase from *Salmonella enterica*, and endogenous pantothenate kinase. The resulting strain produced 3-HP at 1.0 g/L from a culture starting at a glucose concentration of 50 g/L. We also engineered the sugar supply by displaying beta-glucosidase (BGL) on the yeast cell surface. When grown on 50 g/L cellobiose, the beta-glucosidase-displaying strain consumed cellobiose efficiently and produced 3-HP at 3.5 g/L. Under fed-batch conditions starting from cellobiose, this strain produced 3-HP at up to 11.4 g/L, corresponding to a yield of 11.2% (g-3-HP/g-glucose; given that 1 g cellobiose corresponds to 1.1 g glucose upon digestion).

**Conclusions:**

In this study, we constructed a series of *S. pombe* strains that produced 3-HP via the malonyl-CoA pathway. Our study also demonstrated that BGL display using cellobiose and/or cello-oligosaccharides as a carbon source has the potential to improve the titer and yield of malonyl-CoA- and acetyl-CoA-derived compounds.

**Electronic supplementary material:**

The online version of this article (10.1186/s12934-018-1025-5) contains supplementary material, which is available to authorized users.

## Background

3-Hydoroxypropionic acid (3-HP) is a platform chemical that serves as a precursor to various valuable chemicals, including acrylic acid, acrylonitrile, and 1,3-propanediol. Acrylic acid, which is used as a super absorbent polymer and in plastic manufacturing, currently is produced primarily by catalytic oxidation of petroleum-derived propane. Acrylonitrile, a compound that is produced at more than 14 billion pounds per year, is used to generate plastics, resins, and fibers [1]. Hence, the US Department of Energy has designated 3-HP as one of the most important substances that should be targeted for production from renewable resources [2]. Establishment of 3-HP production from renewable resources such as glucose would provide a bio-sustainable alternative to the production of acrylic acid from fossil resources.

Microbial bioproduction is one of the best solutions for building a sustainable economy. Four pathways (including those employing glycerol, lactate, malonyl-CoA, or β-alanine) have been defined for the production of 3-HP in vivo using microorganisms. *Klebsiella pneumoniae* and *Lactobacillus reutei* are known to produce 3-HP from glycerol [3]. The mechanism of 3-HP production from glycerol consists of 2 steps. First, glycerol is converted to 3-hydroxypropionaldehyde (3-HPA) via a reaction catalyzed by a coenzyme vitamin B12-dependent glycerol dehydratase. Next, 3-HPA is converted to 3-HP via a reaction catalyzed by aldehyde dehydrogenase [4–7]. This route is not suitable for industrial use, because typical host production microorganisms [notably, *E. coli* and budding yeast (*Saccharomyces cerevisiae*)] cannot synthesize vitamin B12; such host organisms therefore would require a continuous exogenous supply of this cofactor [3, 8]. The 3-HP production pathway from lactic acid is thermodynamically unfavorable [9]. Additionally, this reaction yields a mixture of lactate and 3-HP, a combination that is not suitable for industrial use [9]. Both the malonyl-CoA and the β-alanine pathways for 3-HP synthesis employ thermodynamically favorable reactions that do not require coenzyme vitamin B12, making these pathways appealing for industrial use [10–13]. Notably, the NADPH-dependent malonyl-CoA reductase (MCR) from *Chloroflexus aurantiacus* is widely used for 3-HP synthesis. Although MCR catalyzes a two-step reaction of malonyl-CoA to 3-HP via malonate semialdehyde [14], the imbalance of this cascade causes the accumulation of malonate semialdehyde, resulting in a low rate of conversion from malonyl-CoA to 3-HP. Separation of the MCR enzyme into two individual fragments [MCR-C (amino acids 550-1219) and MCR-N (amino acids 1-549)] has been shown to facilitate the rebalancing of the activities of MCR-C and MCR-N, yielding a drastic increase in 3-HP production (3.72 g/L in shaking flask cultivation) when expressed in *E. coli* [15].

Using acid-tolerant host cells is one of the most important techniques for making 3-HP production an economical process. A bacterial host such as *E. coli* or *K. pneumoniae* need pH control during fermentation, and recovery of the desired product subsequently requires acidification of the culture. Acid-tolerant hosts, such as yeasts, have the advantage that the acid form of 3-HP can be produced directly [11]. Notably, budding yeast has been engineered for 3-HP production [16]. However, the growth of *S. cerevisiae* is severely impaired in the presence of 50 g/L 3-HP [16]. Alternatively, *Schizosaccharomyces pombe* is naturally tolerant to 3-HP and can grow even in the presence of 50 g/L 3-HP [16]. However, there are only a few reports of 3-HP production using *S. pombe* as a host [13].

Improvement of precursor supply is necessary for chemical production. Acetyl-CoA is a precursor of 3-HP and of a wide range of bioproducts, including isoprenoids, fatty acids and lipids, and butanol. Several strategies have been reported for enhancing the level of acetyl-CoA in the yeast cytosol, including introduction of a PDH bypass [10, 18], blocking the glyoxylate pathway to decrease acetyl-CoA consumption [19], and enhancing the supply of CoA [20]. In addition, supplying of other key metabolites for bioproduct production can be used to optimize the cultivation process. For instance, the Iriana and Nielsen groups successfully demonstrated 3-HP production using *S. cerevisiae* as a host with defined medium or feed-in-time medium [11]. This feed-in-time medium provides carbon-limited cultivation, possibly because the medium contains high levels of polysaccharides in combination with cellulolytic enzymes, which gradually degrade the polysaccharide and release glucose [11]. The titer of 3-HP on feed-in-time medium was high; however, this *S. cerevisiae* medium has to be specially synthesized and so is not readily available. In contrast, we have developed cell surface-display techniques for this purpose [21–23]. One of our constructs consists of a beta-glucosidase (BGL)-displaying *S. pombe* strain; this strain degrades cellobiose and provides glucose as a carbon source, permitting direct growth on cellobiose without the need for enzymatic supplementation [23]. However, there are no reports (to our knowledge) of the production of acetyl-CoA- and malonyl-CoA-derived chemicals using BGL-displaying *S. pombe*.

**Fig. 1:**
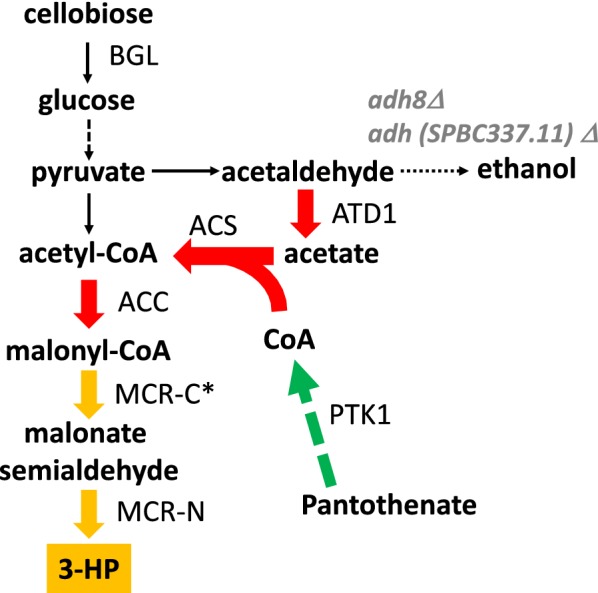
Engineered metabolic pathway for 3-HP production by *S. pombe*. Two genes (*adh8* and *adh* SPBC337,11) were disrupted; the expression of three endogenous genes (*atd1*, *acc*, *ptk1*) was enhanced; and four exogenous genes (acsSE* from *Salmonella enterica*, separate fragments encoding MCR-C* and MCR-N from *Chloroflexus aurantiacus*, and *BGL* from *Aspergillus aculeatus*) were introduced

The present study aimed to improve the 3-HP productivity of an engineered *S. pombe* strain via introduction of a malonyl-CoA pathway. To construct such a 3-HP-producing *S. pombe* strain, we used three strategies: (a) balancing the activity between MCR-C and MCR-N, (b) engineering the supply of acetyl-CoA and CoA itself by metabolic engineering, and (c) engineering the supply of sugar by cell surface engineering (Fig. 1). Our results demonstrate the potential of *S. pombe* for 3-HP production, showing the potential of cello-oligosaccharides as a raw material for 3-HP production.

## Materials and methods

### Strains and media

All strains used in this study are listed in Table 1. *S. pombe* FY12804 (NBRP) was used as the parental strain. Yeast strains were cultivated in YM medium (BD Diagnostic Systems, Sparks, MD, USA) or EMM medium (ForMedium, Norfolk, United Kingdom) supplemented with glucose or cellobiose as a carbon source.

**Table 1:** Strains and plasmids used in this study

Strain	Genotype	Reference/source
12804	*h90 ura4*-*D18 ade6*-*M210 leu1*-*32*	YGRC/NBRP
12804 ku70Δ	*h90 ura4*-*D18 ade6*-*M210 leu1*-*32 ku70Δ*	Ozaki et al. [17]
SPO-01	*12804 ku70Δ, isp6Δ::Pcam1*-*acc*, integration of *mcr* gene at *leu*	This study
SPO-02	*12804 ku70Δ, isp6Δ::Pcam1*-*acc, ppp16Δ::Pef1a*-*c*-*mcr*-C*, adh8Δ::Pef1-*a*-*c*-*mcr*-C*-*Phsp*-*mcr*-*N*	This study
SPO-03	SPO-02, *atg4Δ::Pcam1*-acsSE*, adh4Δ::Pcam1-*atd1*	This study
SPO-04	SPO-03, integration of *Pcam1*-*ptk1* at *leu*	This study
SPO-05	SPO-04, *gut2Δ::Phsp*-*SPBC359.04c*-*BGL*	This study
SPO-06	SPO-05, *aap1Δ::Pcam1*-*atd1, fma2Δ::Pcam1*-acsSE*	This study
SPO-07	SPO-06, *sxa2Δ::Pef1a*-*c*-mcrC*	This study

Plasmid	Description	Reference/source
pDUAL-FFH31	Vector under *cam1* promoter control	RIKEN BRC [37]
pDUAL-FFH61	Vector under *ef1*-*a*-*c* promoter control	RIKEN BRC [37]
pDUAL-FFH1	Vector under *nmt1* promoter control	RIKEN BRC [37]
pDUAL-hsp-SPBC359.04c-BGL	Vector for cell surface-display expression of protein fusion of BGL from *Aspergillus aculeatus* with SPBC359.04c anchor protein	Ozaki et al. [17]
pDUAL-FFH1-MCR	Vector for expression of *mcr* from *Chloroflexus aurantiacus*	This study
pDUAL-FFH61-MCR-C	Vector for expression of *mcr*-C* from *Chloroflexus aurantiacus*	This study
pDUAL-hsp-MCR-N	Vector for expression of *mcr*-*N* from *Chloroflexus aurantiacus*	This study
pDUAL-FFH31-ACC	Vector for expression of *cut6* from *Schizosaccharomyces pombe*	This study
pDUAL-FFH31-ACS	Vector for expression of acsSE* from *Salmonella enterica*	This study
pDUAL-FFH31-ACS1	Vector for expression of *acs1* from *Schizosaccharomyces pombe*	This study
pDUAL-FFH31-ATD1	Vector for expression of *atd1* from *Schizosaccharomyces pombe*	This study
pDUAL-FFH31-PTK1	Vector for expression of *ptk1* from *Schizosaccharomyces pombe*	This study
pMZ374	Vector for expressing *adh1*:*cas9*/*rrk1*:sgRNA in fission yeast: empty sgRNA target	Addgene (#59896)
pMZ374-aap1-1266	pMZ374 derivative, *aap1(1266)* sgRNA target	This study
pMZ374-atg4-46	pMZ374 derivative, *atg4(46)* sgRNA target	This study
pMZ374-fma2-950	pMZ374 derivative, *fma2(950)* sgRNA target	This study
pMZ374-isp6-1069	pMZ374 derivative, *isp6(1069)* sgRNA target	This study
pMZ374-ppp16-83	pMZ374 derivative, *ppp16(83)* sgRNA target	This study
pMZ374-sxa2-1436	pMZ374 derivative, *sxa2(1436)* sgRNA target	This study
pMZ374-adh4-100	pMZ374 derivative, *adh4(100)* sgRNA target	This study
pMZ374-adh8-434	pMZ374 derivative, *adh8(434)* sgRNA target	Ozaki et al. [17]
pMZ374-gut2-1749	pMZ374 derivative, *gut2(1749)* sgRNA target	Ozaki et al. [17]

### Plasmid and homologous recombination (HR) donor construction

Plasmids used in this study are listed in Table 1, and primers are listed in Additional file 1: Table S1. Cas9 and gRNA expression plasmid pMZ374 [24] was purchased from Addgene. A 20-base seed sequence together with the NGG PAM sequence (N20NGG) in the *S. pombe* genome was selected using CRISPR direct (http://crispr.dbcls.jp/) [25]. The HR donor sequences used as editing templates were designed to have about 500-bp homology arms to either side (upstream and downstream) flanking the Cas9 cutting site and the sites of the intended insertions.

A Cas9 expression plasmid targeting the protease-encoding *aap* gene (bp 1-1266) was constructed using the KOD-Plus-mutagenesis Kit (TOYOBO, Co., Ltd., Osaka, Japan) according to the manufacturer’s instructions. pMZ374 was used as a template with the primer pair F-aap1-1266 + R-aap1-1266. The resultant plasmid was named pMZ374-aap1-1266. Other plasmids for Cas9 targeting were constructed in a similar way and are summarized in Table 1.

To construct the MCR expression cassette, the MCR-encoding gene from *Chloroflexus aurantiacus* was amplified by PCR using codon-optimized synthetic oligonucleotide (Invitrogen) as a template with the primer pair For-MCR + Rev-MCR. The PCR product was cloned into the *Nhe*I and *Sal*I sites of pDUAL-FFH1 (RIKEN BRC), and the resultant plasmid was named pDUAL-FFH1-MCR. Other plasmids for expression of *mcr*-c*, acc, acs from *Salmonella enterica* (acsSE*), *atd1*, and *ptk*1 were constructed similarly; these plasmids are summarized in Table 1. The PCR templates for fragments encoding *mcr*-c* and *acs* from *S. enterica* were obtained as codon-optimized synthetic oligonucleotides (Invitrogen). The PCR products encoding *acc*, acsSE*, *atd1*, and *ptk1* were cloned (separately) into pDUAL-FFH-31(RIKEN BRC); the PCR product encoding *mcr*-c* was cloned into pDUAL-FFH-61(RIKEN BRC). The PCR product encoding *mcr*-*n* was cloned between the *Nco*I and *Xho*I sites of pDUAL-hsp-SPBC359.04c_BGL [17]. The resulting plasmid carries *mcr*-*N* under the control of the *hsp* promoter [17].

To construct HR donor DNA for introduction of the *acc* expression cassette into the chromosomal *isp6* region, the *acc* expression cassette (*Pcam*-*acc*-*Tadh1*) was amplified by PCR with the primer pair For-isp6-1069-3 + Rev-isp6-1069-4. Upstream and downstream regions were amplified with the primer pairs For-isp6-1069-1 + Rev-isp6-1069-2 and For-isp6-1069-5 + Rev-isp6-1069-6, respectively. The three amplified fragments were conjugated by overlap extension PCR with the primer pair For-isp6-1069-1 + Rev-isp6-1069-6, resulting in HR donor DNA for introduction of the *acc* expression cassette into the chromosomal *isp6* region. Other HR donor DNAs for introduction of fragments encoding *mcr*-c*, mcr-n, acc, acsSE*, atd1, ptk1, and *bgl* into the respective genomic regions were constructed similarly; these plasmids are summarized in Table 1.

### Genome editing

All mutagenesis experiments were carried out by co-transformation of 10 µL of the pMZ374 vector series with 20 μL of the respective PCR product being used as the HR donor DNA for the target locus, as described in a previous report [17]. Briefly, strains were grown in 5 mL of YM medium at 30 °C with shaking at 180 rpm until the culture reached an optical density (OD) at 600 nm of 0.5; cells then were co-transformed by electroporation using a Gene Pulser Xcell II (Bio-Rad) and standard methodologies. Transformants were selected using EMM+Leu plate, then screened by colony PCR and DNA sequencing.

### Cultivation and analytical methods

For 3-HP fermentation from glucose or cellobiose, strains were pre-cultured in 5 mL YM medium for 3 days at 30 °C with shaking in a 15-mL test tube, then washed twice with 1% NaCl and diluted to an initial OD of 3.0 in 5 mL EMM medium supplemented with appropriate amino acids and containing glucose (50 g/L) or cellobiose (50 g/L or 20 g/L). The resulting cultures were incubated at 30 °C with shaking, under aerobic conditions, with sampling at the indicated time points. Cell growth, glucose, and cellobiose levels were analyzed as described previously (Ozaki et al. [17]). 3-HP and acetic acid levels were analyzed using an HPLC equipped with a SCR-102H column (7 µm, 8.0 mm ID × 300 mm; Shimadzu) as described previously [17].

### Fermentations

SPO-07 was pre-cultured in 5 mL YM medium for 1 day; the preculture then was inoculated into 100 mL YM medium in a 1-L baffled flask and cultured for approximately 24 h. Cells were collected by centrifugation at 900*g* for 10 min and used to inoculate 400 mL EMM medium containing cellobiose as a carbon source in a 1-L jar fermenter. Fermentation was performed as follows: agitation rate, 400 rpm; temperature, 30 °C; aeration, 1 L/min with sterile air; pH 5.0, maintained by automatic addition of NH_3_(aq); dissolved oxygen (DO), 40%, controlled by agitation. For fed-batch cultivations, the feed was started after 24 h of cultivation. The feed contained 200 g/L cellobiose as a carbon source. The feed rate was set to 5 mL/h; a total feed volume of 0.2 L was used per reactor. In the case of glucose was used as a carbon source, initial concentration was 20 g/L and the feed contained 200 g/L glucose as a carbon source. Other conditions were the same as described above. 3-HP and metabolite analyses were as described above.

## Results and discussion

### Reconstruction of a pathway for 3-HP synthesis via malonyl-CoA by coexpression of MCR-C* and MCR-N

To develop a *S. pombe* strain capable of malonyl-CoA-mediated production of 3-HP, we introduced two genes: *C. aurantiacus mcr*, which encodes malonyl-CoA reductase (MCR); and the endogenous *acc* (*cut6)* gene, which encodes acetyl-CoA/biotin carboxylase. MCR converts malonyl-CoA to 3-HP via malonate semialdehyde. ACC has a critical role in both malonyl-CoA supply and fatty acid biosynthesis. Earlier work suggested that strains deficient for intracellular proteases may exhibit improved protein expression [26, 27], a property that might also enhance 3-HP production. In the present work, *mcr* was introduced by complementation of a Leu- auxotrophy with a *leu* marker and *cut6* (expressed under the control of *ef1a*-*c* constitutive promoter) was introduced by gene replacement at the protease-encoding *isp* locus. After a 48-h cultivation in EMM medium supplemented with 50 g/L glucose, a wild-type *S. pombe* strain did not produce 3-HP to a detectable level, whereas the *mcr acc* strain (designated SPO-01) generated 3-HP to a titer of 0.016 g/L (Fig. 1a).

**Fig. 2:**
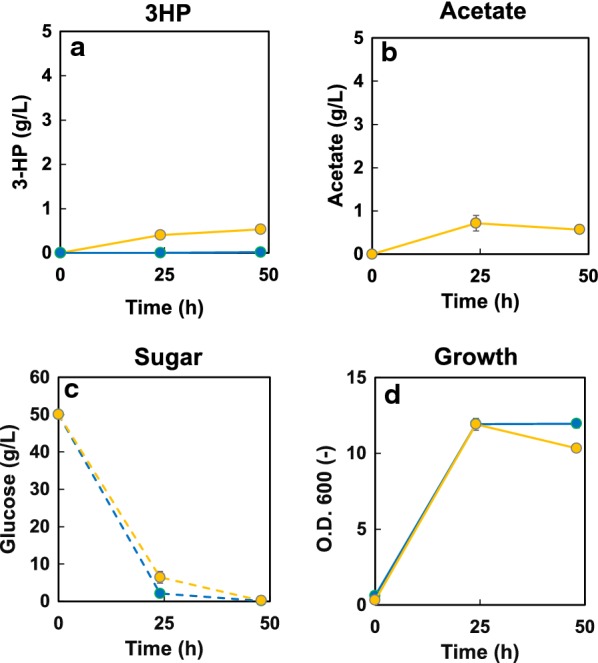
3-HP production from EMM medium containing 50 g/L glucose using the strains SPO-01 and SPO-02. Time courses of 3-HP (**a**), acetate (**b**), sugar (**c**), and growth (**d**) are shown. Strain SPO-01 is represented by blue symbols, SPO-02 by yellow symbols. Separate cultures were started from each of three separate colonies of the respective strains; the resulting data are presented as the mean ± standard deviation from these triplicate cultures

The *C. aurantiacus* MCR is a bi-functional enzyme that catalyzes a two-step reduction from malonyl-CoA to 3-HP. This enzyme can be separated into two fragments, MCR-C and MCR-N (i.e., the C- and N-terminal subdomains). The MCR-C fragment reduces malonyl-CoA to malonate semialdehyde, while the MCR-N fragment reduces malonate semialdehyde to 3-HP [15]. The activities of MCR-C and MCR-N are unbalanced, such that malonyl-CoA reduction by MCR-C is the rate-limiting aspect of the two-step reaction [15]. In *E. coli*, this imbalance was minimized by increasing the expression ratio of MCR-C/MCR-N after saturation mutagenesis of MCR-C, yielding a mutant enzyme referred to here as MCR-C*. To recreate the example from *E. coli*, we introduced both fragments [one encoding MCR-N (*mcrN*) and the second encoding MCR-C* (mcrC*)] into the genome of ACC-expressing *S. pombe*. The resultant strain generated 3-HP to a titer of 0.18 g/L, which was improved compared to SPO-01 (data not shown). We then introduced an additional copy of mcrC*. The resultant strain (SPO-02) harbors 2 copies of mcrC* under the control of *ef1a*-*c* promoter, 1 copy of *mcrN* under the control of the *hsp* promoter, and 1 copy of *acc* under the control of the *cam1* constitutive promoter. SPO-02 generated 3-HP to a titer of 0.53 g/L (Fig. 2a), a 30-fold improvement compared to SPO-01. Both strains consumed almost all of the supplied glucose after 24 h, producing 0.5 g/L of acetic acid as a by-product. Introduction of the *mcrN* and mcrC* fragments did not adversely affect cell growth (Fig. 2d). Introduction of a third copy of mcrC* into SPO-02 did not further enhance 3-HP production (data not shown), suggesting that further improvement of 3-HP titer would require the malonyl-CoA and acetyl-CoA supply.

### Improvement of 3-HP production by increasing the supply of acetyl-CoA

To increase the malonyl-CoA supply, we sought to enhance the acetyl-CoA supply. Notably, engineering of the availability of acetyl-CoA has been reported to yield a positive effect on 3-HP production, presumably via the increase in the supply of malonyl-CoA. This strategy has been employed successfully in *S. cerevisiae* to improve the production of 3-HP [11, 28], butanol [29, 30], and isoprenoids [31, 32]. Specifically, these laboratories enhanced the flux towards acetyl-CoA by co-expression of a *Salmonella enterica* gene encoding acetyl-CoA synthase harboring a point mutation (referred to here as acsSE*) and a gene encoding an intact aldehyde dehydrogenase (*S. cerevisiae ald6*). To apply this approach in *S. pombe*, we introduced acsSE* (under the control of the *cam1* promoter) into the protease-encoding *atg4* locus of strain SPO-02. We then introduced a copy of the endogenous *atd1* gene (a *S. pombe* homolog of the *S. cerevisiae ald6* aldehyde dehydrogenase; also under the control of the *cam1* promoter) into the *adh4* locus (which encodes an alcohol dehydrogenase) of the resulting strain. We chose to target an alcohol dehydrogenase-encoding gene based on the observation that the loss of alcohol dehydrogenase activity permits accumulation of acetoaldehyde, a substrate of aldehyde dehydrogenase. However, given a previous report [17] that disruption of the major *S. pombe* alcohol dehydrogenase locus *adh1* significantly impaired growth, we chose to target the *adh4* locus, which encodes a minor alcohol dehydrogenase.

**Fig. 3:**
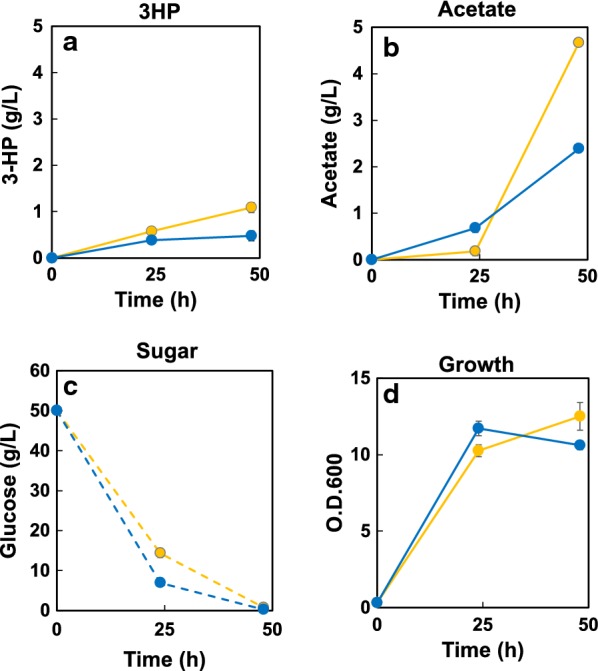
3-HP production from EMM medium containing 50 g/L glucose using the strains SPO-03 and SPO-04. Time courses of 3-HP (**a**), acetate (**b**), sugar (**c**), and growth (**d**) are shown. Strain SPO-03 is represented by blue symbols, SPO-04 by yellow symbols. Separate cultures were started from each of three separate colonies of the respective strains; the resulting data are presented as the mean ± standard deviation from these triplicate cultures

The resulting SPO-03 strain (which harbors acsSE* and *atd1*) did not exhibit an improved 3-HP titer (compared to strain SPO-02) (Fig. 3a). However, SPO-03 accumulated acetate to 2.4 g/L after 48 h of cultivation (Fig. 3b). Introduction of additional copies of acsSE* or of the endogenous *acs* gene (*acs1*) could not yield improved 3-HP production (data not shown).

Engineering the CoA supply is an alternative to for improving the acetyl-CoA supply. Pantothenate kinase (PTK) is a key enzyme for improving CoA production [33]. For example, the overexpression of *mpanK1b* (encoding a mouse PTK homolog) yielded a 13-fold increase of the intracellular CoA concentration in mammalian cells [34]. Similarly, a fivefold increase of acetyl-CoA levels (with tenfold higher CoA production) was obtained by overexpression of the endogenous PTK in *E. coli* [35]. In *S. cerevisiae*, overexpression of the endogenous PTK in combination with the introduction of *acs* and *ald6* was reported to yield ~3.6-fold increased production of naringenin (a flavonoid) compared to the level obtained upon introduction of *acs* and *ald6* alone [20]. To implement this strategy, we introduced a copy of the endogenous gene encoding pantothenate kinase (*ptk1*) into SPO-03 by *leu* auxotrophic marker. The resultant strain (SPO-04) produced 3-HP of 1.1 g/L in after 48 h cultivation (Fig. 3a), a doubling of titer compared to SPO-03. These results indicated that improving the CoA supply is an effective approach for enhancing the production of malonyl-CoA-derived chemicals. Although intracellular amount of acetyl-CoA could not be detected by acetyl-CoA kit (data not shown), acetate accumulation at 48 h was increased from 2.4 g/L (SPO-03) to 4.7 g/L (SPO-04) (Fig. 3b; yellow) in the new strain. The introduction of additional copies of ptk1 did not further improve 3-HP production (data not shown), suggesting that acetyl-CoA is sufficient in strain SPO-04.

### Improvement of 3-HP production by controlling the carbon supply

**Fig. 4:**
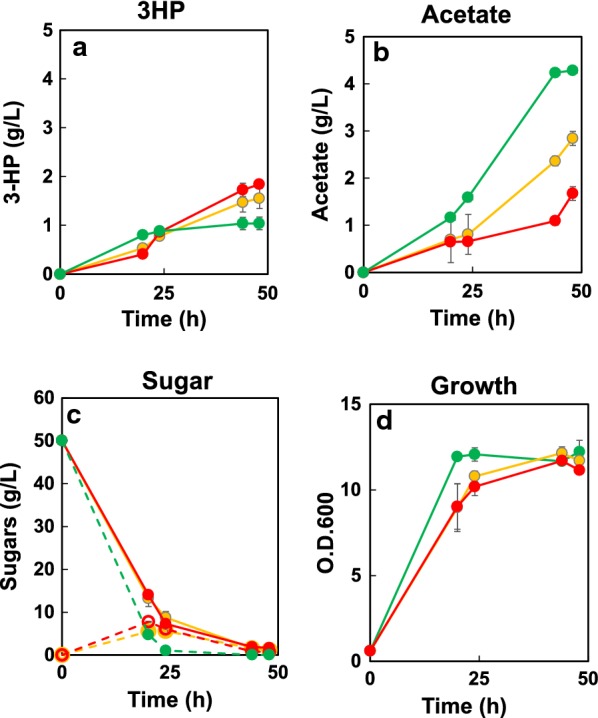
3-HP production from EMM medium containing 50 g/L glucose or cellobiose using the strains SPO-05 and SPO-06. Time courses of 3-HP (**a**), acetate (**b**), sugar (**c**), and growth (**d**) are shown. In **c**, dotted lines show glucose and solid lines show cellobiose. Strain SPO-05 grown on glucose is represented by green symbols; SPO-05 grown on cellobiose is represented by yellow symbols; and SPO-06 grown on cellobiose is represented by red symbols. Separate cultures were started from each of three separate colonies of the respective strains; the resulting data are presented as the mean ± standard deviation from these triplicate cultures

Avoiding ethanol accumulation is a challenge for 3-HP production in budding yeast. Disruption of the ethanol synthesis pathway is a straightforward approach, but halts growth. Alternatively, 3-HP production under carbon-limited cultivation has been demonstrated in *S. cerevisiae* using feed-in-time medium, which imitates a fed-batch process [12]. This feed-in-time medium contains polysaccharides as well as a degrading enzyme, providing a gradual release of glucose. Here, we utilized our previously described cell surface-display technique for producing BGL on the surface of *S. pombe* [23]. BGL was expressed as a genetic fusion with an anchor protein, SPBC359.04c [23]. The resulting BGL localizes to the cell surface, where the enzyme hydrolyzes cellobiose into glucose. Therefore, we introduced the gene encoding the BGL-anchor fusion protein into SPO-04. The resultant strain (SPO-05) produced 3-HP at 1.0 g/L starting from 50 g/L of glucose (Fig. 4a), a titer similar to that obtained with SPO-04. That is the expression of BGL had no adverse effect on 3-HP production. When 50 g/L of cellobiose was used as a carbon source, the BGL-displaying SPO-05 strain produced 3-HP at 1.6 g/L after 48 h of cultivation, a titer was 1.5-fold that obtained with glucose. These results showed that providing cellobiose (or the cello-oligosaccharide form) as the carbon source permits an on-going release of glucose by gradual degradation via the cell surface-displayed BGL, and cell surface display was effective for 3-HP production. During growth on glucose, strain SPO-05 consumed 45.3 g/L of glucose after 20 h; almost all glucose was consumed by 24 h. After 20 h of cultivation on cellobiose, cellobiose and glucose were 14 g/L and 7.7 g/L (respectively) in the medium, corresponding to the consumption of 28.3 g/L of cellobiose. The residual amounts of cellobiose and glucose after 24 h of cultivation were 7.2 g/L and 6.0 g/L, respectively. Thus, SPO-05 cultivated in cellobiose-containing medium appeared to maintain a culture medium glucose concentration of approximately 6–7 g/L. Cell growth on cellobiose was slightly slower than that on glucose, presumably due to slower glucose supply. Acetate accumulation was notably decreased (from 4.2 to 2.8 g/L after 48 h) when cellobiose was used as the carbon source. Maintaining a lower glucose concentration in the culture medium by use of BGL display may prevent overflow metabolism, resulting in reducing acetate accumulation [36].

In an attempt to further improve 3-HP production, we introduced additional copies of *acc* and acsSE* into SPO-05. The resultant strain (SPO-06) produced 3-HP at 1.8 g/L of after 48 h of cultivation, a slight improvement compared to SPO-05. Although SPO-06 exhibited decreased acetate accumulation (1.6 g/L in 48 h cultivation), 3-HP production was not improved. This observation suggested that an improved malonyl-CoA supply was failed to enhance 3-HP production. Therefore, further improvement of MCR activity is desirable.

### Re-tuning of MCR-C expression level

**Fig. 5:**
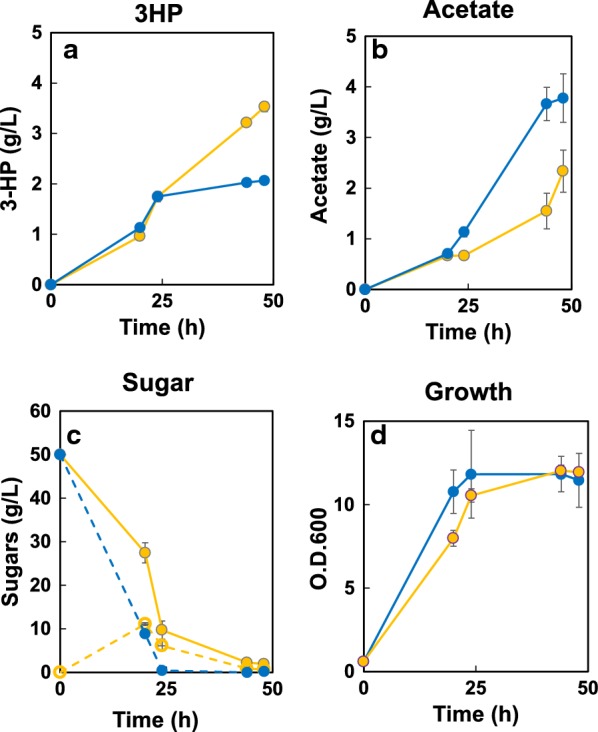
3-HP production from EMM medium containing 50 g/L glucose or cellobiose using strain SPO-07. Time courses of 3-HP (**a**), acetate (**b**), sugar (**c**), and growth (**d**) are shown. In **c**, dotted lines show glucose and solid lines show cellobiose. Strain SPO-07 grown on glucose is represented by blue symbols; SPO-07 grown on cellobiose is represented by yellow symbols. Separate cultures were started from each of three separate colonies of the respective strains; the resulting data are presented as the mean ± standard deviation from these triplicate cultures

MCR activity was improved in the SPO-02 strain, and the acetyl-CoA supply then was further strengthened in the SPO-04 strain. By employing cellobiose as a carbon source with BGL display, 3-HP titer was augmented to 1.8 g/L in the strain SPO-05. Further enhancement of the flux toward acetyl-CoA decreased acetate accumulation, and the resultant strain SPO-06 exhibited a slightly improved 3-HP titer. Remarkably, SPO-06 exhibited a significantly strengthened malonyl-CoA supply compared to that in SPO-02. Hence, we anticipated that the further augment of MCR activity (to utilize the increased malonyl-CoA supply) might further enhance 3-HP production. Hence, additional copies of the mcrC* gene were inserted into the chromosomal *sxa2* locus of SPO-06. It is difficult to evaluate the activities of mcrC and mcrN because both enzymes use NADPH as a cofactor. However, enzyme expression was confirmed by SDS-PAGE (Additional file 1: Figure S1A) and qPCR analysis of the resulting strain (SPO-07) revealed that the relative mRNA expression level of mcrC* increased with gene copy number (Additional file 1: Figure S1B). Figure 5 shows that SPO-07, which carries 3 copies of mcrC* in its genome, produced 2.0 g/L of 3-HP from 50 g/L glucose after 48 h of cultivation, a titer twofold higher than that obtained with SPO-05 (1.0 g/L of 3-HP from 50 g/L of glucose). When cellobiose (50 g/L) was used as the carbon source, SPO-07 produced 3-HP at 3.5 g/L after 48 h of cultivation, a titer twofold higher than that obtained with SPO-06 (as described above). This level corresponded to a yield of 0.063 g-3-HP/g-glucose (given that 50 g cellobiose corresponds to 55.5 g glucose upon digestion). Introduction of a 4-copies of *mcr*-C* or 2-copies of *mcr*-*N* did not further increase the 3-HP titer (data not shown).

### Fed-batch cultivation using cellobiose as a carbon source

**Fig. 6:**
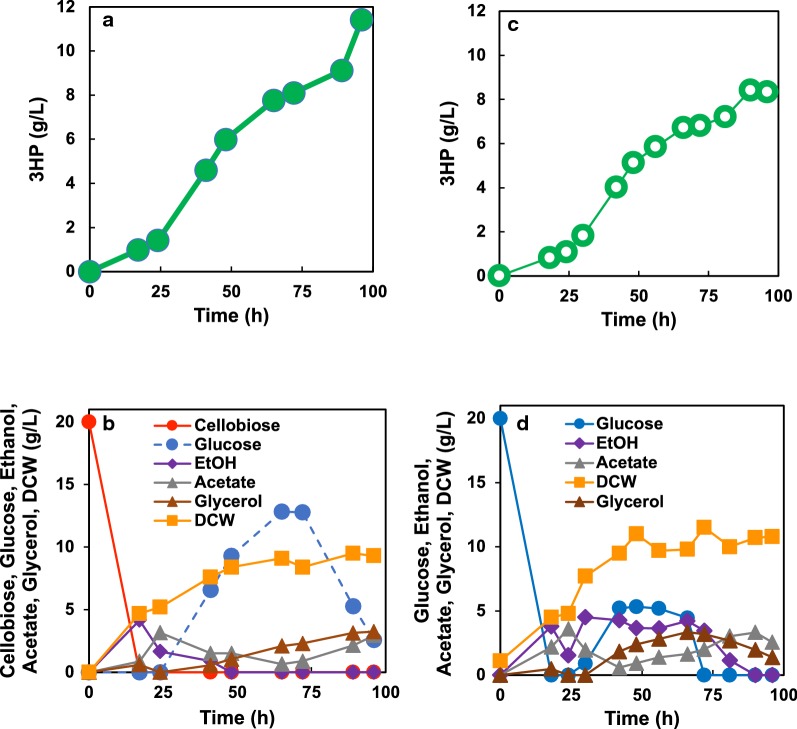
Fed-batch cultivation of strain SPO-07 using cellobiose (**a**, **b**) or glucose (**c**, **d**) as a carbon source. Time courses of 3-HP (**a**, **c**), other metabolites and dry cell weight (DCW) (**b**, **d**) are shown. The concentration of cellobiose (red circles), of glucose generated from cellobiose by cell surface BGL (dotted blue circles), of ethanol (purple triangles), acetate (gray triangles), glycerol (brown triangles), and DCW (orange squares) are shown. In **d**, glucose concentration is shown solid blue line. The cultivations were performed in triplicate (Additional file 1: Figure S2); representative results are shown here

Production of 3-HP under aerobic fed-batch conditions with the limited feeding of cellobiose was evaluated. Specifically, EMM medium supplemented with cellobiose as a carbon source was used. After 24 h of cultivation, when almost all of the original cellobiose was exhausted, further cellobiose feeding was initiated. Under these conditions, strain SPO-07 produced 11.2 ± 0.6 g/L of 3-HP (Fig. 6a) after 96 h of cultivation, corresponding to a yield of 0.12 ± 0.007 g-3-HP/g-glucose. The volumetric production rate in the fed-batch phase was 0.14 g/L/h, corresponding to a specific yield of 1.22 g/g-dry cell weight (DCW). Low levels of acetate and glycerol accumulated under fed-batch culture conditions (Fig. 6b). Glucose concentration increased gradually from the start of feeding, and virtually all of the remaining residual glucose was consumed after feeding was stopped (after 65 h). Notably, the results reported here [titer 11.2 ± 0.6 g/L, yield 12 ± 0.7% (g-3-HP/g-glucose; 1 g cellobiose is equivalent to 1.1 g glucose)] are superior to those previously reported for malonyl-CoA-dependent 3-HP production using *S. cerevisiae*. When glucose was used as a carbon source, 3-HP production under fed-batch conditions was also evaluated. After 24 h cultivation, when all of the original (20 g/L) glucose was consumed, further glucose (200 g/L of glucose in feeding medium) feeding was started. Strain SPO-07 also produced 9.2 ± 0.9 g/L of 3-HP (Fig. 6c) after 96 h of cultivation, corresponding to a yield of 11 ± 0. 11 g-3-HP/g-glucose. Similarly, low levels of acetate and glycerol accumulated under fed-batch culture conditions (Fig. 6d). During feeding phase, ethanol production was kept at low level in spite of glucose accumulation (Fig. 6c, d). Enhancement of glucose uptake may be one of the ways for improving 3-HP titer, as well as shutoff by-products producing pathways. To our knowledge, this work is the first report of chemical production using beta-glucosidase-displaying fission yeast cells under fed-batch condition with cellobiose or glucose feeding.

## Conclusions

In this study, we constructed a series of *S. pombe* strains that produced 3-HP via the malonyl-CoA pathway. 3-HP production was significantly improved through combinatorial strategies including coordination of the expression of MCR-N and MCR-C, overexpression of the acetyl-CoA synthetic pathway, and enhancement of the CoA synthesis pathway. The additional incorporation of BGL display and the use of cellobiose as a carbon source improved 3-HP titer to 11.2 ± 0.6 g/L, corresponding to a yield of 12 ± 0.07% (g-3-HP/g-glucose; 1 g cellobiose is equivalent to 1.1 g glucose) using fed-batch cultivation with cellobiose feeding. These results were superior to the peak 3-HP production previously reported in budding yeast using the malonyl-CoA pathway. Our study also demonstrated that BGL display using cellobiose and/or cello-oligosaccharides as a carbon source has the potential to improve the titer and yield of malonyl-CoA- and acetyl-CoA-derived compounds.

## Additional file

**Additional file 1.** Additional figures and tables.
