# Supplementary material for: Enhancing 3-hydroxypropionic acid production in combination with sugar supply engineering by cell surface-display and metabolic engineering of Schizosaccharomyces pombe
Source: Microb Cell Fact. 2018 Nov 13;17:176. doi: 10.1186/s12934-018-1025-5 (PMC6234659; doi:10.1186/s12934-018-1025-5)
Supplement: Supplementary file 1 — Additional file 1. Additional figures and tables. [file 12934_2018_1025_MOESM1_ESM.pdf]

Additional File 1

Enhancing 3-hydroxypropionic acid production in combination with sugar supply engineering by cell surface-display and metabolic engineering of *Schizosaccharomyces pombe*

Seiya Takayama[1], Aiko Ozaki[1], Rie Yamaguchi[2], Chisako Otomo[2], Mayumi Kishida[2], Yuuki Hirata[2], Takuya Matsumoto[2], Tsutomu Tanaka[1*] and Akihiko Kondo[2]

[1] Department of Chemical Science and Engineering, Graduate School of Engineering, Kobe University 1-1, Rokkodaicho, Nada, Kobe 657-8501, Japan

[2] Graduate School of Science, Technology and Innovation, Kobe University, 1-1 Rokkodaicho, Nada, Kobe 657-8501, Japan

Corresponding author: Tsutomu Tanaka

Tel/Fax: +81-78-803-6202

e-mail: tanaka@kitty.kobe-u.ac.jp

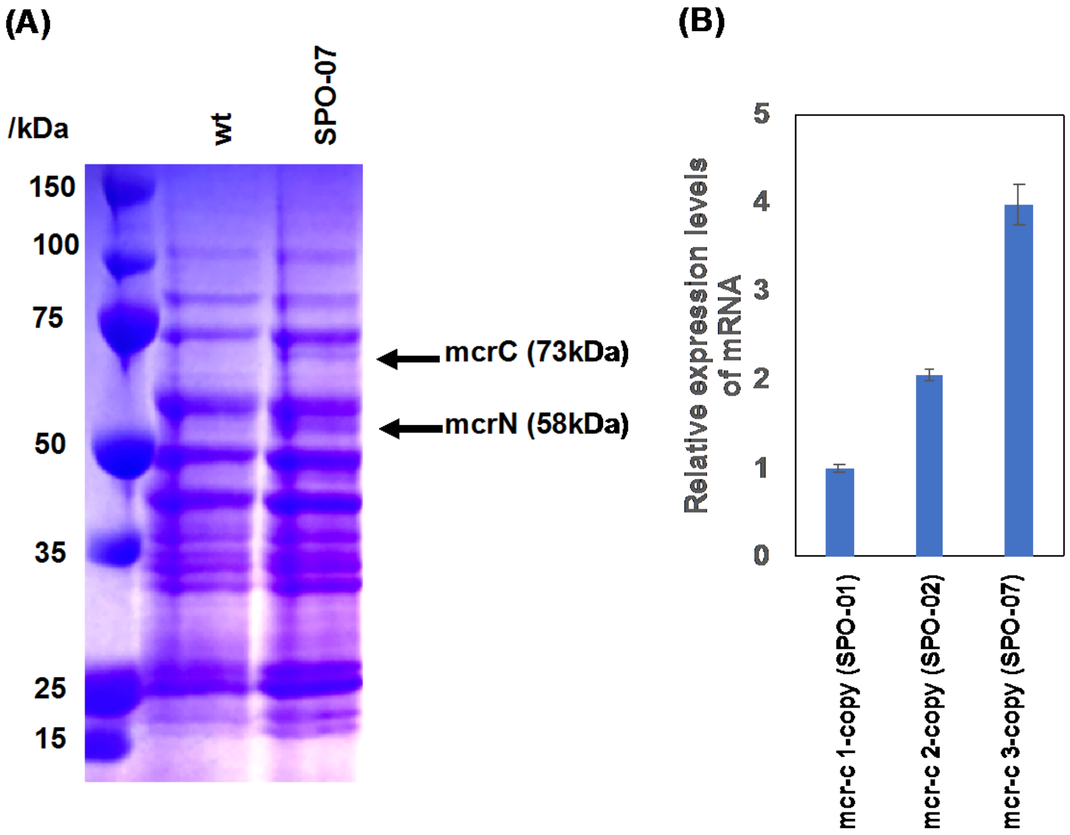

Figure S1. (A) SDS-PAGE analysis of wild type strain and SPO-07 strain.

(B) Relative transcriptional levels of MCR-C in SPO-01, SPO-02, SPO-07. The mRNA was extracted using NucleoSpin RNA kit (TAKARABIO). RT-PCR was carried out using RNA-direct SYBR Green Realtime PCR Master Mix (TOYOBO) with the MCR-C primer pair of 5'-CCTAATCGTGCTGATTATGCTG-3' and 5'-GTTCTCCAGTACCTCTAAGACGATC-3'. His3 was used as a standard with the primer pair of 5'-GCTACCATTATGAACTCATTAAAGGCTCC-3' and 5'-TCTGTGCCAACAGTGATACGCAATGCTCC-3'

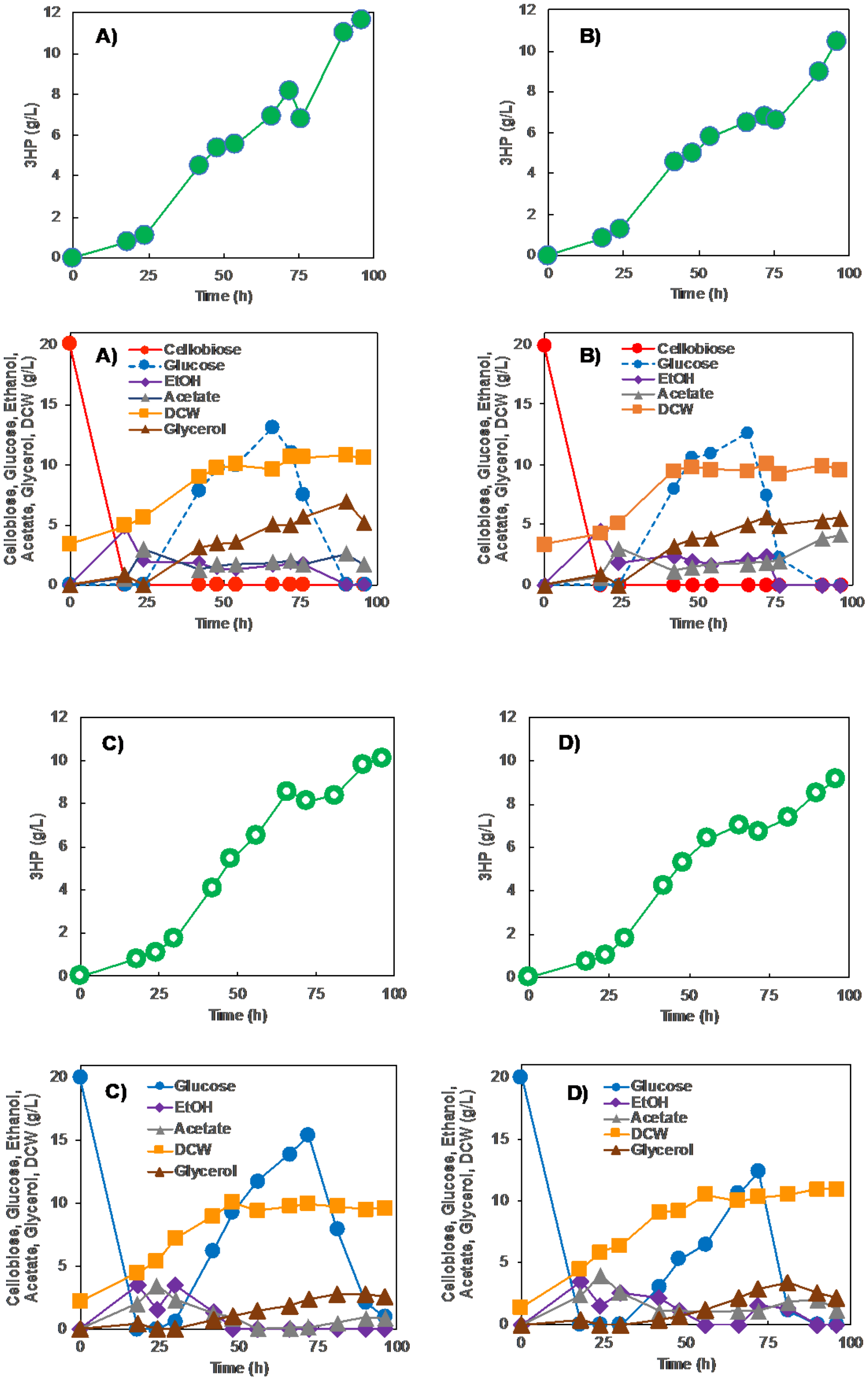
A)
3HP (g/L)
Time (h)
B)
3HP (g/L)
Time (h)
A)
Cellobiose
Glucose
EtOH
Acetate
DCW
Glycerol
Cellobiose, Glucose, Ethanol, Acetate, Glycerol, DCW (g/L)
Time (h)
B)
Cellobiose
Glucose
EtOH
Acetate
DCW
Cellobiose, Glucose, Ethanol, Acetate, Glycerol, DCW (g/L)
Time (h)
C)
3HP (g/L)
Time (h)
D)
3HP (g/L)
Time (h)
C)
Glucose
EtOH
Acetate
DCW
Glycerol
Cellobiose, Glucose, Ethanol, Acetate, Glycerol, DCW (g/L)
Time (h)
D)
Glucose
EtOH
Acetate
DCW
Glycerol
Cellobiose, Glucose, Ethanol, Acetate, Glycerol, DCW (g/L)
Time (h)

Figure S2. Fed-batch cultivation of strain SPO-07 using cellobiose (A and B) or glucose (C and D) as a carbon source. Time courses of 3-HP (A, C), other metabolites and dry cell weight (DCW) (B, D) are shown. The cultivations were performed in triplicates; here the replicates of fermentations from Figure 6 are shown.

Table S1. Primers used in this study

For-acc	AGTTACTGATGCTAGCATGCGCCAGAGCGTTACCTC
Rev-acc	ATCCAGGCCTGTCGACTTAATTAACCGACGCGAGTT
For-acs	AGTTACTGATGCTAGCATGTCTCAAACTCATAAGCA
Rev-acs	ATCCAGGCCTGTCGACAGAAGGCATAGCAATAGCTT
For-atd1	AGTTACTGATGCTAGCATGTCTACAAAACTCGTTGA
Rev-atd1	ATCCAGGCCTGTCGACTTAAATGGGAGAATCCATTC
For-ptk1	AGTTACTGATGCTAGCATGAGTGAAACAGAATGCGG
Rev-ptk1	ATCCAGGCCTGTCGACCTACGATATTGAGGGAACAG
For-mcr	CCTCTTCAAGTTTAAGAATCGTTTAGAGCTAGCATGTCTGGTACTGGACGTTTAGCTGG
Re-mcr	CCATGATATCCGGATCCAGGCCTGTCGACTTAAACAGTAATAGCTCTTCCACGATGAATA
For-mcrC	ATCGTTTAGAGCTAGCATGTCTGCTACTACAGGAGCTAGTCTGCTTCTG
Rev-mcrC	ATCCAGGCCTGTCGACTTAAACAGTAATAGCTCTTCCACGATGAATACG
For-mcrN	AATATTCAAACTCGAGATGTCTGGTACTGGA
Rev-mcrN	ACATTCCTTGCGGCCGCTTAAATATTAGCAGGAAT
For-aap1-1266-1	AATATGGAAACAGAATTTGT
Rev-aap1-1266-2	ACAACGTAGTATGCTATAGCATGAGCCATGTCAGCAGAAA

For-aap1-1266-3	TTTCTGCTGACATGGCTCATGCTATAGCATACTACGTTGT
Rev-aap1-1266-4	CGACTGCATACTCAAAGTCCATATTACCCTGTTATCCCTA
For-aap1-1266-5	TAGGGATAACAGGGTAATATGGACTTTGAGTATGCAGTCG
Rev-aap1-1266-6	TATTTAATCTTTTCTTTCGT
For-atg4-46-1	ATAATGTTACCTATCACTAATATAGCTCAT
Rev-atg4-46-2	AGTATGCTATAGCGCAGGCTCGGTGTTTGTCGGTG
For-atg4-46-3	ACAAACACCGAGCCTGCGCTATAGCATACTACGTT
Rev-atg4-46-4	AAACCAAATTAATGTTATATTACCCTGTTATCCCT
For-atg4-46-5	TAACAGGGTAATATA ACATTAATTTGGTTTTTAGG
Rev-atg4-46-6	ACAAGAACAAGATGTTGTAGGTCCAAACCA
For-isp6-1069-1	GATACCGGTGTAAGCATTCATCATGTTGAG
Rev-isp6-1069-2	AGTATGCTATAGCGCGGTGATGGCTTTCTTAGAAG
For-isp6-1069-3	AAGAAAGCCATCACCGCGCTATAGCATACTACGTT
Rev-isp6-1069-4	GTTTATAGTGGAAGCTATATTACCCTGTTATCCCT

For-isp6-1069-5	TAACAGGGTAATATAGCTTCCACTATAAACGACCA
Rev-isp6-1069-6	CTATTCTTGAGCACCATTGAAAGCGAGAAG
For-fma2-950-1	TTATTAAGCCCGGCATGTCT
Rev-fma2-950-2	TCACAAAGATGAGTCCGGTTGTTTTTCCGTTAATTTCCAC
For-fma2-950-3	GTGGAAATTAACGGAAAAACAACCGGACTCATCTTTGTGA
Rev-fma2-950-4	ATTCGCCAATTCTGTCCAAGATATTACCCTGTTATCCCTA
For-fma2-950-5	TAGGGATAACAGGGTAATATCTTGGACAGAATTGGCGAAT
Rev-fma2-950-6	CAATAGTATGGAAATACGAG
For-sxa2-1436-1	TTCCACAATATCTGAAGAGT
Rev-sxa2-1436-2	TCTCCTAAAATGATTGAAATAGAAAGACACTTTGTATTTT
For-sxa2-1436-3	AAAATACAAAGTGTCTTTCTATTTCAATCATTTTAGGAGA

Rev-sxa2-1435-4	ATAAACGATAGTTATTTATTATATTACCCTGTTATCCCTA
For-sxa2-1436-5	TAGGGATAACAGGGTAATATAATAAATAACTATCGTTTAT
Rev-sxa2-1436-6	TGATCCTTACCGTCATATTG
For-adh4-1	TTGGAGGTGATTTGATGTCGTTTGGAAAAG
Rev-adh4-2	AATGCATGCAAGCTTATCGGTAACAATTAAGGTGT
For-adh4-3	TTAATTGTTACCGATAAGCTTGCATGCATTTCAAT
Rev-adh4-4	ACCGACCTTAATAATCGTTGTAAAACGACGG
For-adh4-5	CGTCGTTTTACAACGATTATTAAGGTCGGTTTGTA
Rev-adh4-6	GGGATTAGCAGCAGTAGAGACGTAAGCTTC
For-adh8-1	ATGGCTTTGCGATACGTTGTTCATGATGAA
Rev-adh8-2	AGTATGCTATAGCGCCGCTGTTACGGCTGCGCATG
For-adh8-3	GCAGCCGTAACAGCGGCGCTATAGCATACTACGTT
Rev-adh8-4	TCCAAACAACCCATTACTAGTATATTACCCTGTTA
For-adh8-5	GGGTAATATACTAGTAATGGGTTGTTTGGATCCAA
Rev-adh8-6	AAACACCTTGTCAACCACCGGGTGAATGTC
F-aap1-1266	GCCAAAAAACATAACCTGTACCGAAGAATGCCAATTCAGTCATTCGGTTTA GAGCTAGAAATAGCAAGTTAAAATAAGGCTAGTCC
R-aap1-1266	GGACTAGCCTTATTTTAACTTGCTATTTCTAGCTCTAAAACAATGACTGAAT

	TGGCACATTCTTCGGTACAGGTTATGTTTTTTGGC
F-atg4-46	GCCAAAAAACATAACCTGTACCGAAGAACCGACAAACACCGAGCCTCCGTTTTAGAGCTAGAAATAGCAAGTTAAAATAAGGCTAGTCC
R-atg4-46	GGACTAGCCTTATTTTAACTTGCTATTTCTAGCTCTAAAACGGAGGCTCGGTGTTTGTCGGTTCTTCGGTACAGGTTATGTTTTTTGGC
F-fma2-950	GCCAAAAAACATAACCTGTACCGAAGAACGTGGACAACACCACGTCGTTTTAGAGCTAGAAATAGCAAGTTAAAATAAGGCTAGTCC
R-fma2-950	GGACTAGCCTTATTTTAACTTGCTATTTCTAGCTCTAAAACCGTGGTGTTGTCCACGAATTCTTCGGTACAGGTTATGTTTTTTGGC
F-isp6-1069	GCCAAAAAACATAACCTGTACCGAAGAATCTAAGAAAGCCATCACCGTGTTTTAGAGCTAGAAATAGCAAGTTAAAATAAGGCTAGTCC
R- isp6-1069	GGACTAGCCTTATTTTAACTTGCTATTTCTAGCTCTAAAACACGGTGATGGCTTTCTTAGATTCTTCGGTACAGGTTATGTTTTTTGGC
F-sxa2-1436	GCCAAAAAACATAACCTGTACCGAAGAATGATCCTACTGGATCTACGTTTTAGAGCTAGAAATAGCAAGTTAAAATAAGGCTAGTCC
R-sxa2-1436	GGACTAGCCTTATTTTAACTTGCTATTTCTAGCTCTAAAACAGATCCAGTAGGATCAAGTTCTTCGGTACAGGTTATGTTTTTTGGC
F-adh4-100	GCCAAAAAACATAACCTGTACCGAAGAAACCTTAATTGTTACCGATCCGTTTTAGAGCTAGAAATAGCAAGTTAAAATAAGGCTAGTCC
F-adh4-100	GGACTAGCCTTATTTTAACTTGCTATTTCTAGCTCTAAAACGGATCGGTAACAATTAAGGTTTCTTCGGTACAGGTTATGTTTTTTGGC
